# Inducible HIV RNA transcription assays to measure HIV persistence: pros and cons of a compromise

**DOI:** 10.1186/s12977-017-0385-y

**Published:** 2018-01-17

**Authors:** Johann Plantin, Marta Massanella, Nicolas Chomont

**Affiliations:** 1 Department of Microbiology, Infectiology and Immunology, Faculté de Médecine, Université de Montréal, Montréal, QC Canada; 2 Centre de Recherche du CHUM, Université de Montréal, 900 rue St-Denis, H2X0A9 Montréal, QC Canada

**Keywords:** HIV reservoir, Induced RNA, HIV transcription

## Abstract

With the increasing number of therapeutic strategies tested in humans to reduce the size of the latent reservoir, the development of a robust, precise and clinical trial scalable assay that measures the frequency of infected cells carrying inducible replication-competent HIV is urgently needed. The size of the pool of cells carrying replication-competent HIV is largely overestimated by DNA assays, as a result of a large proportion of defective viruses, and underestimated by co-culture outgrowth assays. New culture methods that measure the inducible HIV reservoir have been developed during the past few years. In these induction assays, CD4^+^ T cells from virally suppressed individuals are activated and HIV RNA is measured in cell extracts or cell supernatants. In this review, we summarize the principle and outcomes of these assays and discuss the potential of these methods in the evaluation of HIV eradication strategies.

## Introduction

In virally suppressed individuals on antiretroviral therapy (ART), only about one in a million resting CD4^+^ T cells contains latent proviruses capable of producing replication-competent HIV [1–4]. Quantifying the frequency of cells harbouring persistent HIV is critical to evaluating strategies to eliminate them, but the low frequency of these cells makes this extremely challenging.

The frequency of HIV-infected cells can be measured using PCR-based assays to determine the frequency of cells harbouring total or integrated HIV DNA [1, 5–8]. These are the quickest and easiest assays to measure the frequency of HIV-infected cells, but they largely overestimate the size of the reservoir by detecting defective proviruses [1]. Culture-based assays such as the quantitative viral outgrowth assay (QVOA), which measure the functional reservoir, have been traditionally used to detect outgrowth of replication-competent virus [2]. However, QVOA underestimates the size of the HIV reservoir due to suboptimal induction and/or inadequate propagation of all replication-competent viruses in vitro [9]. Furthermore, despite its relevance, QVOA is time-consuming, labor-intensive, expensive and requires a large number of cells.

New culture methods that measure the inducible HIV reservoir have been proposed as alternate assays to estimate the size of the HIV reservoir. In most of these assays, resting or total CD4^+^ T cells from virally suppressed individuals are activated in the presence of antiretrovirals (ARVs) and HIV RNA is directly measured from cell extracts (cell-associated RNA, ca-RNA) [10–14] or cell supernatants (cell-free RNA, cf-RNA) [10, 13, 15]. An intrinsic limitation of these assays is the fact that while the production of ca-RNA reflects transcriptionally competent provirus, it also detects defective genomes that can be partially or entirely transcribed. Similarly, quantification of cf-RNA reflects the capacity to generate and release viral particles but does not assess their replication-competency.

The format (bulk vs. limiting dilution) of these inducible RNA-assays can be adapted to answer specific questions. For instance, a limiting dilution can be employed to measure the frequency of CD4^+^ T cells carrying inducible HIV genomes [10, 13, 14], whereas bulk activation of CD4^+^ T cells, which is much less expensive and cumbersome, is useful to determine dose/response curves of latency-reversing agents (LRAs) [10, 15–17]. A limitation of bulk assays is the proliferation of infected cells induced by stimulation (especially in long-term cultures), which may lead to the production of ca-RNA and/or cf-RNA by daughter cells and to an overestimation of the inducible reservoir. This proliferation bias can be corrected by measuring the level of expression of host cellular RNAs, such as ribosomal RNAs or housekeeping genes.

Another significant advantage of inducible HIV RNA assays is that they do not rely on a virus propagation step by co-culture, which may be a limiting step of the QVOA. In this review, we summarize the knowledge gained from the results generated by several ca-RNA and cf-RNA assays and highlight the advantages and limitations of these methods (summarized in Table 1).

**Table 1:** Characteristics of assays measuring the inducible HIV reservoir

References	Format^a^ and application^b^	Target transcript		Stimulation method	Stimulation time (hours)	RT-ddPCR/RT-qPCR/scdPCR^c^	Required CD4 T cells/assay (million)	Advantages	Limitations
		cfRNA	caRNA						
Procopio et al. [14]	LD RQ + LRA	–	tat/Rev	PMA + ionomycin	12	RT-qPCR	< 1	Fast, simple, robust and sensitive method, with no RNA extraction and small amounts of cells required Measures frequency of cells expressing msRNA	Does not evaluate RNA levels produced in individual cell
Yukl et al. [11]	B LRA	–	Profiling panel	aCD3/CD28 + IL-2	24–48	RT-ddPCR	6–10	Exhaustive analysis of the modulation of the transcriptional profile upon activation Enables the identification of blocks HIV transcription, elongation and splicing after stimulation	Does not evaluate RNA levels produced in individual cell
Bullen et al. [17]	B LRA	poly-A	Profiling panel	PMA + ionomycin or LRA	6–18	RT-qPCR	5–10	Analysis of the modulation of the transcriptional profile upon activation Enables the quantification of mature HIV transcripts Adapted to evaluate the activity of LRAs	Does not evaluate RNA levels produced in individual cell
Massanella et al. [13]	LD RQ + LRA	gag	gag tat/rev	aCD3/CD28	72	RT-ddPCR	4.5–9	Combines the value of frequency based assays with cell-associated/cell-free assays Great potential for translational analysis from provirus to virion detection Measures frequency of cells expressing msRNA	Labour intensive Does not evaluate RNA levels produced in individual cell
Cillo et al. [10]	LD RQ + LRA	pol	pol	aCD3/CD28or SAHA	168	RT-qPCR	12	Combines the value of frequency based assays with cell-associated/cell-free assays Good potential for translational analysis from provirus to virion detection	pol RNA increases chances of measuring defective proviruses Labour intensive Does not evaluate RNA levels produced in individual cell
Fromentin et al. [15]	B RQ + LRA	LTR-gag	–	aCD3/CD28 or LRA	168	RT-qPCR	5	Fast and simple Adapted to evaluate the activity of LRAs	Does not evaluate RNA levels produced in individual cell
Yucha et al. [12]	B + SC RQ + LRA	–	gag tat/rev	aCD3/CD28 or LRA	18	scdPCR/RT-ddPCR/RT-qPCR	0.5–1	Allows characterization and quantification of transcriptionally active cells in response to various stimuli High-throughput and reduced costs compared to other single-cell platforms Can be adapted to other HIV transcripts Enables the characterization of reservoir cells by rescuing genomic and viral DNA as well as mRNA	Technically demanding

^a^Format. *LD* Limiting dilution, *B* bulk, *SC* single-cells

^b^Application. *RQ* Reservoir quantification, *LRA* Assessment of LRA potency

^c^*RT-ddPCR* reverse transcription digital droplet PCR, *RT-qPCR* quantitative reverse transcription real-time PCR, *scdPCR* single-cell-in-droplet PCR

Quantification of ca- and cf- RNA is usually performed using single round or semi-nested quantitative reverse transcription real-time PCR (RT-qPCR). The use of semi-nested RT-qPCR enables accurate RNA quantification in samples with a low number of RNA copies and with increased quantitative range compared to single round qPCR assays [18]. In both techniques, serial dilutions of plasmid DNA or synthetic RNA molecules covering the region of interest can be used as standards. An important advantage of RNA standards is that they take into account the efficiency of the reverse transcription step, which can be a limiting step in these assays. More recently, reverse transcription digital droplet PCR (RT-ddPCR) has been proposed as an alternative method to traditional RT-qPCR for absolute quantification of HIV RNA [19], as it allows absolute quantification using Poisson statistics without the requirement for a standard curve. While RT-ddPCR does provide absolute quantification of target DNA, its application to absolute quantification of RNA may be more problematic due to the lack of internal controls for reverse transcription. The use of standard RNA as in RT-qPCR can facilitate higher accuracy of RNA quantification with RT-ddPCR. Importantly, there is a good correlation between RT-ddPCR and semi-nested RT-qPCR for ca-RNA quantification [20]. Both methods show equally high detection rates of unspliced RNA (usRNA) and multiply spliced RNA (msRNA) [20].

Another important technical aspect to take into account when performing PCR-based assays to detect HIV transcripts is the possibility of sequence variation in the target sequence between different individuals. This limitation is particularly important for assays that measure msRNA (tat/rev), a highly variable region of the HIV genome [14, 21], and probably less of a concern for assays measuring more conserved regions such as the gag or pol gene [21].

There is a significant heterogeneity in the nature of the defect within the pool of defective proviruses. For instance, individuals treated at the chronic phase of HIV infection show a striking 98% of defective proviruses, with 80% of defects being internal deletions at different genomic locations. Interestingly, individuals treated in the acute phase of infection have similarly high frequencies of defective genomes but present more hypermutations and fewer deletions compared to chronic-phase treated individuals [22]. This underlines another important consideration when using HIV PCR based assays, which is the fact that the variations between study groups such as time before treatment will be of importance as it appears to alter sequence conservation and defect patterns in different ways.

## Assays that measure inducible cell-associated HIV RNA

Assays that measure inducible ca-RNA usually include the potent activation of CD4^+^ T cells by anti-CD3/CD28 antibodies [10–13], phorbol 12-myristate 13-acetate (PMA) and ionomycin [14] or LRA [10, 12, 17] followed by quantification of HIV RNA by ultrasensitive PCR specific for a given viral transcript. Different RNA species can be quantified using specific PCR methods. These include usRNA [10, 17], msRNA (i.e. tat/rev) [12, 14, 18], poly-A tailed mature RNA transcripts [17, 23], TAR RNAs [11, 24] and the chimeric host-HIV read-through transcripts [17, 25]. The format of the assay (bulk [11, 12, 17], limiting dilution [10, 13, 14] or even single cell [12]) offers different angles of approach to address specific questions, including the frequency and the transcriptional status of latently infected cells as well as the proportion of transcriptionally competent genomes. Consequently, each method introduces limitations that restrict the use of each assay to specific questions.

The Tat/rev Induced Limiting Dilution Assay (TILDA) [14] was developed as a proxy to measure the frequency of blood CD4^+^ T cells carrying inducible latent HIV. In brief, CD4^+^ T cells are stimulated with PMA/ionomycin in the presence of ARVs, counted and plated in limiting dilutions, and msRNA transcripts (tat/rev) are measured by ultrasensitive quantitative RT-qPCR. In virally suppressed HIV-infected individuals, TILDA measured a median frequency of 24 cells expressing msRNA per million CD4^+^ T cells after induction, which correlates with several PCR-based assays for HIV DNA. Interestingly, these frequencies are almost 50 times higher than those measured in QVOA, the gold standard for measuring replication-competent HIV, and 6–27 times lower than the frequencies of infected cells carrying HIV DNA measured by PCR-based assays. [14]. TILDA measures tat/rev transcripts that are required (but not sufficient) for the production of viral particles. Because tat/rev transcripts are generated after splicing of full-length viral transcripts, TILDA reduces the likelihood of measuring proviruses with large internal deletions, which is not the case for other PCR-based assays. On the other hand, TILDA does not bypass the possibility of measuring defective proviruses since infected cells may produce tat/rev transcripts, but might still lack the ability to produce infectious viral particles due to other defects outside the tat/rev region [22]. Nevertheless, a large fraction of defective viruses appear to have deletions encompassing the tat/rev region [9]. TILDA presents several advantages: it is robust and sensitive in measuring the frequency of cells harbouring inducible provirus, it requires as little as 10 mL of blood (less than 1 million CD4^+^ T cells) and can be completed in 2 days. Therefore, TILDA may be very useful in clinical research to monitor the efficacy of therapeutic strategies aimed at reducing the size of the latent HIV reservoir.

The term “leaky latency” has emerged to describe a state of incomplete transcriptional latency. Cells harbouring HIV genomes may transcribe low levels of short and elongated viral RNA and may also produce minute levels of viral proteins during ART [26, 27]. It is therefore relevant to determine the transcriptional profile of latently infected cells, as it could give critical insights as to which mechanisms dictate HIV latency. Bullen et al. [17] and Yukl et al. [11] determined the transcriptional profile of CD4^+^ T cells from virally suppressed individuals on ART. Bullen et al. [17] designed an assay to measure the potency of different LRAs compared to PMA/ionomycin stimulation using three types of transcripts (usRNA gag, read-through and mature RNA using the poly-A tail). Interestingly, treatment of CD4^+^ T cells from ART-suppressed individuals with vorinostat induced a concomitant, yet small increase in gag and read-through transcripts. On the other hand, vorinostat had no effect on the levels of polyadenylated RNA suggesting that the drug induced transcription of non-polyadenylated usRNA. In contrast, maximal activation using PMA/ionomycin greatly increased gag usRNA and mature RNA compared to read-through transcripts, reflective of LTR initiated transcription. To identify the inhibitory steps of HIV transcription, Yukl et al. [11] characterized the HIV transcriptional profile of CD4^+^ T cells following stimulation with anti-CD3/CD28 and IL-2 for 2 days. An exhaustive panel of transcripts representing different steps of the transcription process was assessed by ddPCR from extracted RNA. This panel included poly-A (matured), tat/rev (msRNA transcripts), TAR (all transcripts), read-through (transcriptional interference) and long LTR (elongated transcripts). The results revealed that in non-stimulated cells, most of the transcription is blocked at the elongation, maturation and multiple splicing steps. Activation of CD4^+^ T cells led to increased amounts of elongated, mature and msRNA transcripts. Interestingly, although activation drastically increased the absolute amount of msRNA transcripts, the ratio between msRNA and polyadenylated transcripts remained very low even after activation of the cell, revealing that less than 10% of the total matured transcripts belong to the msRNA type. Therefore, the major reversible inhibition steps to HIV expression in latently infected CD4^+^ T cells from individuals on suppressive ART may reside in blocks to elongation, maturation and multiple splicing, and not necessarily in the initiation of transcription. This method could also be used to analyse the transcriptional profile of different subsets of latently infected CD4^+^ T cells, as well as different tissues in which they reside. A limitation of this assay is that these quantifications were performed in bulk populations of cells: it does not provide frequencies of cells that produce these transcripts, and whether the pattern that is seen in the cell population is reflective of individual cells. The assay could, therefore, be adjusted for a replicate terminal cell dilution use in the future to address this particular question. In addition, like other PCR-based assays, this assay does not exclude defective proviruses that can produce different forms of viral transcripts. Although these limitations have to be evaluated, they do not diminish the highly informative value of this study.

By combining quantifications of gag usRNA and tat/rev msRNA transcripts in a limiting dilution format, Massanella et al. [13] measured a frequency of cells in which these viral transcripts can be induced after stimulation with anti-CD3/CD28 antibodies. In accordance with the study by Yukl et al. [11], the median frequency of cells producing gag usRNA was significantly higher than the frequency of cells producing tat/rev msRNA (106.5 cells per million versus 23 cells per million respectively), pointing again at the intrinsic blocks in advanced steps of HIV transcription.

Yucha et al. [12] developed a single cell assay that enables the quantification of HIV transcriptionally reactivated cells by using innovative single-cell-in-droplet PCR (scdPCR). In this technique, single cells were encapsulated into oil droplets containing a master mix (PCR enzymes, primers, probes, and cell lysis agents). Cells were lysed within isolated droplet microenvironments followed by PCR amplification of tat/rev msRNA and usRNA. Droplets containing infected cells can be sorted by positive fluorescence followed by downstream quantification of mRNA as well as human and viral genomic DNA from bulk or single cells. In this study, resting CD4^+^ T cells from individuals on ART were stimulated through the TCR or with the HDACi romidepsin for 18 h and the transcriptional profiles of the proviruses were analysed in bulk or in single cells. Interestingly, there was a certain disjunction between bulk and single cell results following stimulation, for both usRNA and msRNA transcripts: HDACi stimulation led to an increase in bulk usRNA together with a decrease in the number of transcriptionally active cells. Similar discrepancies were reported for msRNA in stimulated and unstimulated contexts. In some samples, there was an increase in the number of cells expressing usRNA, and yet a decrease in the level of usRNA per million cells. Overall the results suggest inter-individual discrepancies between the number of individual cells in which HIV transcription is induced and the amount of ca-RNA levels recorded in bulk assays. Several technical limitations have to be considered: the current assay may occasionally lead to the loss of the packaged cell in the droplet, and more than one cell can potentially be packaged per droplet, which could bias the results. It is also possible that the droplets may incorporate HIV RNA that is not comprised in intact cells, for instance, free RNA or cell debris/fragments. In addition, defective proviruses were not excluded from the analysis and a significant fraction of transcriptionally active cells do not carry replication-competent genomes. Finally, competition or antagonism can occur when multiple assays are performed in the same droplet. Notwithstanding these limitations, this assay has the potential to rescue the genomic DNA and mRNA of isolated cells, enabling their in-depth characterization. Therefore, this method could be used to evaluate the performance of future LRAs in single cells, and evaluate whether the increase in viral ca-RNA following provirus reactivation derives from a small fraction of active cells or from a large pool of less active cells. It is important to note that although it might be feasible in the future, Yucha et al. did not report ca-RNA copy numbers in single cells. Thus, the precise quantity of different ca-RNA transcripts per cell remains to be determined.

To estimate the frequency of latently infected cells, Derdeyn et al. used in situ hybridization (ISH) with anti-HIV-RNA probes combined with microscopy in PBMCs from virally suppressed and viremic individuals before and after 24 h of PHA and IL-2 stimulation [28]. Although the frequency of circulating viral RNA positive cells was extremely low during viral suppression, it was still detectable in 16 out of 18 individuals receiving ART. A higher frequency of inducible ca-RNA was observed in viremic compared to aviremic individuals and correlated with plasma viral loads. Although these experiments are time-consuming since the cells have to be counted using microscopy, ISH remains a sensitive method that can be used to measure very low frequencies of cells expressing ca-RNA. In addition, the assay enables the isolation of individual ISH-positive cells for further sequencing analysis.

Recently, novel flow-cytometry assays combining the concomitant detection of mRNA (gag-pol) by FISH and the gag-p24 protein have been developed [29–32]. Using this assay, Martrus et al. observed that the relative expression and kinetic of HIV RNA and p24 depend on the nature of the stimuli [30]. Grau-Expósito et al. demonstrated that although all CD4^+^ T cell subsets (except naïve) have a marked increase in HIV-RNA after stimulation, the effector memory subset shows the highest proportion of HIV-RNA expression, and express the strongest correlation with plasma viral load [29]. Baxter et al. mostly measured HIV transcription/translation in viremic untreated individuals, as well as in aviremic treated individuals, and found a latent reservoir varying from 1.2 to 660/million HIV^RNA+/gag+^ cells in the latter [32]. Interestingly, this assay correlated relatively well with measured of HIV DNA, yet expressed noticeable differences in the median frequency of reservoir cells compared to integrated HIV DNA and QVOA measures [32]. These assays are extensively discussed in the article by Baxter et al. in this Special Issue [33].

## Assays that measure inducible viral particles

**Fig. 1:**
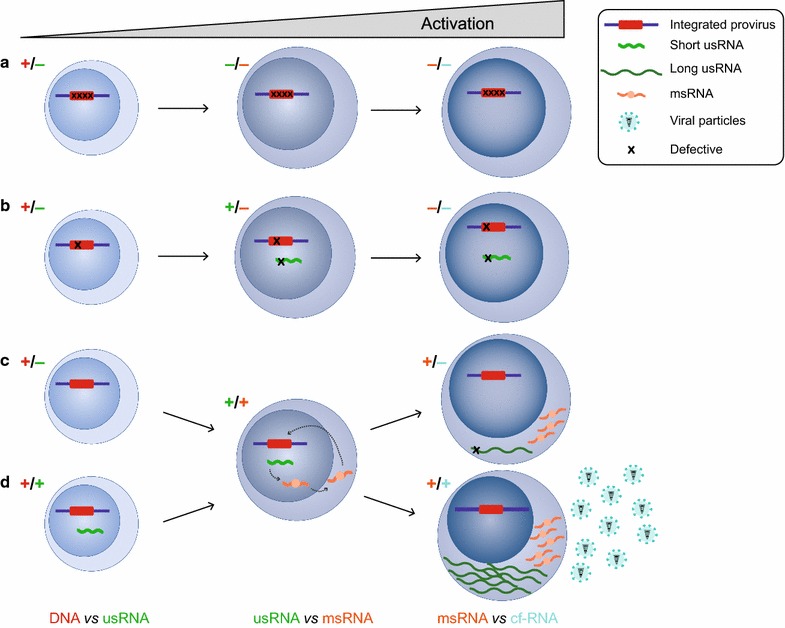
Transcriptional profiles of proviruses after stimulation. **a** A severe defect in the HIV genomes can completely abrogate its ability to produce viral transcripts after induction. **b** Some proviruses can produce short unspliced viral transcripts (usRNA) upon activation, but those may not undergo splicing, preventing the generation of multiply-spliced transcripts (msRNA). **c, d** Intact proviruses can be fully latent (**c**) or partially latent (leaky latency, **d**) After stimulation, msRNA are produced leading to the nuclear export of the full-length viral transcripts that results in the production of viral particles. Colour coded (+) and (−) symbols represent the presence or the absence, respectively of the molecular forms of HIV (DNA [red], usRNA [green], msRNA [orange] or cf-RNA [turquoise]) indicated at the bottom of the figure

Similar to ca-RNA assays, several assays have been developed to measure cf-RNA in culture supernatants after strong stimulation of CD4^+^ T cells in limiting dilution or in bulk. Since the presence of cf-RNA in the culture supernatant reflects the production of new viral particles, cf-RNA assays may be more reflective of the competent HIV reservoir than ca-RNA quantification (Fig. 1). This potential advantage is however mitigated by the fact that not all viral particles are infectious [34] since several defects in the viral genomes will not preclude release of viral particles.

Optimal recovery of viral particles is critical in these assays. Indeed, in addition to viral particles-associated RNA, culture supernatant may contain naked RNA, exosomes, cell fragments/debris and intact cells, which may result in an overestimation of the number of viral particles released. HIV virions can be pelleted by high-speed centrifugation (> 23,000*g* for 1 h), which allows the exclusion of naked RNA, but does not exclude exosomes or contaminating cells. After centrifugation, the RNA is extracted on a column [10, 15] or using TRIZOL [16]. Alternatively, nucleic acid extraction can be performed using more recent techniques such as bead-based RNA extraction, which eliminates the need for ultracentrifugation and efficiently removes potential inhibitors [13], but does not exclude the abovementioned HIV RNA molecules that are not packaged into virions. Once cf-RNA is isolated, HIV RNA can be reverse-transcribed and amplified by PCR using specific primers against conserved regions of the HIV genome, such as gag or pol. Quantification of cf-RNA can be performed by real-time RT-qPCR using a standard curve inferred from a sample with known concentration of viral RNA [10, 15, 16] or by RT-ddPCR which provides an absolute quantification of the target when RT controls are used [13]. The limits of detection of these assays differ depending on the PCR method used, ranging from 3 copies to 150 copies per ml.

The relationship between HIV transcription and virion production has been partially addressed by Massanella et al. [13] and Cillo et al. [10] in different, yet comparable ways. In both studies, the frequencies of cells producing ca-RNA and cf-RNA (viral particles) forms were determined concomitantly. Briefly, resting (Cillo et al.) or total (Massanella et al.) CD4^+^ T cells seeded in limiting dilution were TCR-stimulated using anti-CD3 and anti-CD28 antibodies in the presence of ARV drugs to avoid viral spread. While Cillo et al. pursued the culture for 7 days, Massanella et al. measured viral production after only 3 days. In both assays, cf-RNA from culture supernatant from each well was extracted and viral cf-RNA was quantified. The frequency of infected cells was then estimated based on Poisson statistics, like previously reported for determination of Infectious Units per Million (IUPM) cells in QVOA [35]. A great advantage of both assays is the fact that the frequency of cf-RNA and ca-RNA producing cells can be quantified simultaneously by extracting RNA from culture supernatant or cells, respectively. Cillo et al. [10] showed that on average 7.5% of viral genomes produce usRNA while only 1.5% have the ability to produce detectable levels of cf-RNA after activation [10]. Interestingly, a 7-day stimulation with SAHA revealed very limited induction of cf-RNA (0.079%) compared to TCR stimulation (1.5%) and no correlation between ca-RNA induction and virion production was noted. The authors concluded that SAHA was unable to broadly reactivate HIV transcription, maybe due to its limited effect on factors involved in latency reversal. In a similar study that simultaneously compared cf-RNA and ca-RNA, Massanella et al. [13] found that the frequency of cells producing usRNA gag and msRNA tat/rev are a median of 25- and 5- fold higher than the frequency of cells producing viral particles (cf-RNA). Taken together, these studies indicate that a proportion of transcriptionally competent proviruses (measured as ca-RNA) are unable to generate viral particles (measured as cf-RNA), suggesting sequence defects or post-transcriptional blocks in a substantial proportion of cells harbouring ca-RNA. Massanella et al. further compared the frequency of cells harbouring replication-competent virus by using a modified QVOA [13]. The frequency of cells harbouring replication-competent HIV was only a median of 1.9 fold higher than the frequency of cells producing viral particles (measured by cf-RNA). In 58% of the samples tested, the frequencies determined by QVOA and cf-RNA measurements were equivalent. These results indicate that a large fraction of the virions produced are replication-competent, and suggest that this assay may be used as a surrogate to QVOA.

Fromentin et al. [15] developed the HIV persistence detection assay (HPDA) in which they measured production of viral particles from 5 million TCR-stimulated CD4^+^ T cells after 6 days of culture in the presence of ARV. The culture was performed in 96 deep-well plates (up to 2 mL of culture medium/well), which allows higher viral production, probably as a result of close cell–cell contacts and provides sufficient culture medium for 5 million cells. Viral production was positively correlated with the frequency of cells harbouring integrated and total HIV DNA, induced tat/rev ca-RNA (measured by TILDA) and QVOA. Therefore, HPDA may be a simple, affordable and clinical scalable assay to assess HIV reservoir in individuals on suppressive ART. An important limitation is the relatively long culture period after stimulation (6 days), which may lead to the proliferation of infected cells. This likely leads to increased levels of viral production by daughter cells, which may vary between donors due to different cell proliferation rates.

In addition to viral RNA, novel ultrasensitive assays have the ability to detect and quantify minute amounts of HIV proteins. Using an ultra-sensitive p24 digital assay (Simoa), which is 1000-fold more sensitive than the classical ELISA, Wu et al. [36] observed that inducible p24 levels trend positively with the TILDA readout. However, Passaes et al. [37] showed that HIV RNA production is infrequently accompanied by p24 protein production in culture supernatants of CD4^+^ T cells from ART-suppressed individuals following LRA stimulation. These results point to a limitation in the evaluation of LRA that rely solely on the production of viral particles (cf-RNA) in culture supernatants and suggest that ultrasensitive Gag p24 assay could be used as a complementary marker in the evaluation of efficient viral reactivation strategies.

## Discussion/conclusion

The definitive test of a cure will require interruption of ART; it is necessary, however, to measure the impact of eradication strategies on the size of the viral reservoir even as therapy is continued if clinical trials are to proceed in an efficient and ethical manner.

Viral induction assays may be considered as a reasonable compromise between simple PCR based assays for HIV DNA and complex co-culture assays on several levels. First, viral induction assays are obviously more time-consuming than HIV DNA quantification assays by PCR, but much less cumbersome than QVOA. Although this criterion may be of importance in large cohort studies, it is probably less relevant in small proof-of-concept studies aimed at reducing the size of the reservoir, in which the most relevant assays should be favoured, irrespective of its level of complexity or cost. Nonetheless, co-culture assays that measure replication-competent HIV such as QVOA present other shortcomings, the most important being the fact that they do not capture all cells harbouring replication-competent HIV.

All viral induction assays measure viral forms (either ca-RNA or cf-RNA) that are intermediates between HIV genomes measured by PCR and replication-competent viruses measured by QVOA. The activation step included in these induction assays may be critical, as latent HIV (i.e. non-transcriptionally active) is thought to be the most important obstacle to a cure for HIV infection. Results from the few induction assays already published suggest that the quantification of viral particles in culture supernatants of stimulated CD4^+^ T cells (in limiting dilution or in bulk) may be an acceptable surrogate to replication-competent HIV. This is somewhat surprising as the majority of viral particles are thought to be non-infectious [34], although this result remains controversial [38]. Even more surprising is the observation by Derdeyn et al. [28] who found the frequency of viral RNA-positive cells to be equivalent to the frequency of cells that produce infectious virus. Even though these studies require further validation, they suggest that viral induction assays measuring frequencies of cells producing ca-RNA and cf-RNA may reflect more accurately the frequencies of cells with replication-competent HIV when compared to HIV DNA quantification. If proven true, this would greatly facilitate the measurement of the size of the reservoir by substantially reducing the time of culture.

There are several important limitations of current inducible HIV RNA transcription assays. First, similar to QVOA, they currently all rely on a single round of T cell activation, which may underestimate the frequency of inducible genomes. In addition, the optimal duration of stimulation may vary as a function of the nature of the viral transcript to be quantified and may require further optimization. For instance, longer durations of activation may be required to quantify cf-RNA compared to ca-RNA, but T-cell proliferation (which starts after a single day of activation) may confound interpretation of the results. Finally, all viral induction assays rely on the detection of HIV transcripts by RT-qPCR and the primers and probes may not optimally recognize all viral quasispecies, particularly when samples from individuals infected with non-B clades are analyzed.

In addition to their capacity to measure the size of the viral reservoir, viral induction assays are valuable tools to evaluate the activity of LRAs. This can be done in limiting dilution format [10] to calculate a frequency of cells that get reactivated after a single or a combination of compounds. However, the frequency of cells reactivated by LRAs is usually much lower than the one obtained after maximal stimulation with PMA/ionomycin or anti-CD3/CD28 antibodies. For this reason, activation of bulk CD4^+^ T cells may be a preferred as a first approach in order to reduce the number of cells required and the cost of the assay and would enable testing various concentrations of LRAs or drug combination in a single experiment [15–17].

Viral induction assays represent attractive surrogates to QVOA for the measurement of the replication-competent reservoir. Recent data demonstrating that a substantial fraction of defective proviruses have the ability to produce viral transcript and even proteins [39] suggest that these assays are likely to overestimate the size of the pool of cells harbouring replication-competent HIV. Additional studies are warranted to precisely determine the extent of this overestimation and whether it varies between different individuals. Results from these analyses will help the HIV research community to determine if viral induction assays are suitable methods to evaluate the efficacy of therapeutic strategies aimed at reducing the size of the replication-competent reservoir.
